# Impact of Selenium on Biomarkers and Clinical Aspects Related to Ageing. A Review

**DOI:** 10.3390/biom11101478

**Published:** 2021-10-07

**Authors:** Urban Alehagen, Trine B. Opstad, Jan Alexander, Anders Larsson, Jan Aaseth

**Affiliations:** 1 Division of Cardiovascular Medicine, Department of Medical and Health Sciences, Linköping University, SE-581 85 Linköping, Sweden; 2 Center for Clinical Heart Research, Department of Cardiology, Oslo University Hospital Ullevål, P.O. Box 4950 Nydalen, N-0424 Oslo, Norway; t.b.opstad@medisin.uio.no; 3 Faculty of Medicine, University of Oslo, N-0370 Oslo, Norway; 4 Norwegian Institute of Public Health, P.O. Box 222 Skøyen, N-0213 Oslo, Norway; Jan.Alexander@fhi.no; 5 Department of Medical Sciences, Uppsala University, SE-751 85 Uppsala, Sweden; anders.larsson@medsci.uu.se; 6 Department of Research, Innlandet Hospital Trust, P.O. Box 104, N-2381 Brumunddal, Norway; jaol-aas@online.no; 7 Faculty of Health and Social Sciences, Inland Norway University of Applied Sciences, N-2624 Lillehammer, Norway

**Keywords:** selenium, ageing, cardiovascular, cancer, sirtuins, telomeres

## Abstract

Selenium (Se) is an essential dietary trace element that plays an important role in the prevention of inflammation, cardiovascular diseases, infections, and cancer. Selenoproteins contain selenocysteine in the active center and include, i.a., the enzymes thioredoxin reductases (TXNRD1–3), glutathione peroxidases (GPX1–4 and GPX6) and methionine sulfoxide reductase, involved in immune functions, metabolic homeostasis, and antioxidant defense. Ageing is an inevitable process, which, i.a., involves an imbalance between antioxidative defense and reactive oxygen species (ROS), changes in protein and mitochondrial renewal, telomere attrition, cellular senescence, epigenetic alterations, and stem cell exhaustion. These conditions are associated with mild to moderate inflammation, which always accompanies the process of ageing and age-related diseases. In older individuals, Se, by being a component in protective enzymes, operates by decreasing ROS-mediated inflammation, removing misfolded proteins, decreasing DNA damage, and promoting telomere length. Se-dependent GPX1–4 and TXNRD1–3 directly suppress oxidative stress. Selenoprotein H in the cell nucleus protects DNA, and selenoproteins residing in the endoplasmic reticulum (ER) assist in the removal of misfolded proteins and protection against ER stress. In this review, we highlight the role of adequate Se status for human ageing and prevention of age-related diseases, and further its proposed role in preservation of telomere length in middle-aged and elderly individuals.

## 1. Introduction

Ageing has been described as an imbalance between damage inflicted through the antioxidative defenses of an organism and the harmful production of reactive oxygen species (ROS) [1]. Oxidative damage to biomacromolecules (proteins, nucleic acids, and lipids) accompanying harmful ROS production can represent a condition for developing age-related diseases [2], whereas a programmed part of the ageing process may proceed independently from oxidative stress or external exposures [3].

The trace element selenium (Se), as a significant essential nutritional factor, may remodel biochemical and physiological changes accompanying ageing by improving immune functions, mediating metabolic homeostasis and antioxidant defense, and also in the removal of misfolded proteins [4,5,6]. Se deficiency in ageing populations seems to increase the risk of developing age-related diseases [7,8]. In the EVA study, low levels of Se appeared to decrease human life expectancy by increasing vulnerability to different diseases, suggesting blood Se values to represent a longevity index in an aged population [9]. This French study included 1389 men and women aged around 65 years at inclusion. After the 9-year follow-up period, those who were alive were found to have a higher baseline plasma selenium (1.1 μmol/L) compared with those who had died (1.0 μmol/L) [8]. Maintenance of adequate Se status in the elderly appears to positively affect the self-perception of health, physical activity and quality of life [10,11]. Adequate Se status has been reported to protect against myocardial infarction [12]. Population-based studies have reported that decreased serum Se and total carotenoid concentrations were related to an elevated risk of death among older women in an American population [13]. Of note, it has been observed that fewer individuals with advanced age reside in Chinese counties with endemic Keshan disease, a cardiomyopathy precipitated by Se deficiency, in comparison with counties free of this condition [14], indicating elevated mortality from chronic diseases in Se-deficient areas. In consistence, a recent ecological study from China observed higher distribution ratios of older people living in the eastern and southern coastal regions of the country and one of several factors associated with longevity in these regions was higher soil Se levels [15]. Furthermore, in COVID-19, which seems to be an age-related disease as the lethality increases strongly with age [16], a low selenium status was associated with an unfavorable outcome [17]. Aside from its role in antioxidant defense, Se is known as an important trace element for alleviating metal toxicity [18] and for adequate immune responses [17]. Its presence within at least 25 selenoproteins in the form of the amino acid selenocysteine (SeC) has attracted attention regarding human health [19]. Several researchers have reported that Se deficiency or inadequate supply is an important issue affecting millions of people worldwide [20]. In the case of deficiency, supplementation with organic selenium in the form of selenomethioneine, the main dietary form of selenium [21,22], has frequently been used [23]. 

The aim of the present review is to highlight the importance of an adequate selenium status for healthy aging.

## 2. Selenium Nutrition: From Basic to Clinical Aspects

Se is absorbed into the body by Se-related transporters in the distal part of the small intestine, and after uptake in the liver it may either enter the methionine pool, which may serve as a long-term storage for Se, or it is metabolized to hydrogen selenide and incorporated into selenoprotein P (SELENOP). The latter operates as a circulating selenium carrier, and after uptake in various extrahepatic tissues via low-density lipoprotein receptor-related protein 8 (LRP8), selenocysteine is, by the action of β-lyase, degraded to selenide, from which several other selenoproteins are synthesized, such as glutathione peroxidases (GPXs), thioredoxin reductases (TXNRDs), and methionine sulfoxide reductase [21]. The antioxidative properties of selenoproteins are presumed to have a role in cellular protection in ageing [24]. A widely accepted hypothesis proclaims that the process of ageing is paralleled by an oxidation-reduction imbalance characterized by excessive production of ROS and/or reduced ROS scavenging, resulting in impaired cellular functions and cell senescence [25]. Antioxidants, such as the Se-containing GPXs and TXNRDs, assist in lowering free radical reactants to “tolerable” levels [26]. The Se-dependent glutathione peroxidases (GPX1–4 and GPX6) remove peroxides, and thioredoxin reductases (TXNRD1–3) play a central role in cellular redox regulation, thereby suppressing oxidative stress, hence being essential for cell survival [27,28]. Methionine sulfoxide reductase 1 reduces oxidized sulfur in methionine residues of proteins. Dietary adequacy of Se is proposed to be critical for maintaining adequate redox functions in cells and tissues [7]. Recently, it has been revealed that Se can decrease DNA damage in the leukocytes of hemodialysis patients; and although not directly extrapolative to humans, selenite may prolong telomere lengths in cells in vitro, and by such mechanisms possibly slow down the ageing process [24]. Supplementation with Se combined with coenzyme Q_10_ given to elderly individuals with low values of both was able to prevent or alleviate age-associated diseases, such as cardiovascular diseases (CVDs) and neuropsychiatric disorders [29,30].

Ageing is related to changes in the renewal of proteins and mitochondria, and the aggregation of misfolded proteins seems to be a central feature in ageing and in age-related diseases, such as Alzheimer’s disease and type 2 diabetes [31,32]. Several selenoproteins, i.a., selenoproteins F, K, S and T, which reside in the endoplasmic reticulum (ER), appear to participate in the control and removal of misfolded proteins and in the protection against ER stress, also including control of calcium homeostasis [4,5,33]. Hence, adequate selenium status may play a role in healthy ageing by these mechanisms also.

There are few studies in experimental animals on the role of selenium in ageing. However, contrary to other studies discussed in this review showing an apparent positive impact of selenium on healthy ageing, a recent study in mice found that selenium deficiency did not reduce lifespan despite a dramatic reduction in selenoprotein expression [34]. Extensive characterization of metabolic changes induced by selenium deficiency indicates that the changes showed similarities to changes associated with pro-longevity related to nutrient sensing. In an earlier study in mice, selenium deprivation prolonged lifespan but caused increased signs of senescence and impaired age-related health [35]. Increased lifespan and less age-related pathology were observed in mice that were heterozygous knockout for GPX4 [36].

Several studies have been carried out concerning ageing and environmental and nutritional factors, including Se consumption, particularly in Chinese areas. In longevity areas in China (five provinces), Se concentrations were highest in the oldest person who showed a median value of plasma Se of 1.4 μmol/L, which is well above the level needed for the full expression of selenoproteins. The contents of plasma Se, iron, and copper (Cu) in centenarians were higher than in those aged around 90 [37]. An ecological study of all 18 counties and cities of the Hainan Province showed a positive association between the daily intake of Se from water and food and indexes of ageing and longevity [38]. 

## 3. Selenium Deficiency—A Role in Diseases in the Elderly 

### 3.1. Ageing and Inflammation

The consequences of aging include numerous changes at the cellular and molecular levels. Among the most characteristic features in the ageing process are increased expression of acute phase reactants, such as C-reactive protein (CRP), tumor necrosis factor alpha (TNF-α), and interleukin-6 (IL-6) [39]. 

Mitochondrial injuries appear to be an important factor in cellular senescence. The free radical theory of aging states that the generation and leakage of ROS from the mitochondrial respiratory chain increases with age, leading to cellular oxidative damage [25]. Apparently, oxidative stress, inflammation, and ageing interact with each other in a complex way, and inflammation accompanying ageing is often referred to as “inflammaging” [40]. Inflammation in the elderly is considered to represent a risk factor for several diseases, including CVD, cancer, and dementia [41,42,43]. Supplementation of Se in vivo appears to enhance antioxidant capacity and alleviate inflammation [44]. Among inflammatory biomarkers evaluated in a cohort of elderly individuals supplemented with a combination of Se and coenzyme Q_10_ in the Swedish KiSel project [12], the markers CRP, P-selectin [45] and osteoprotegerin (OPG) [46] were reduced or normalized following a four-year period of supplementation. In another cohort encompassing elderly women, Se supplementation was observed to ameliorate obesity-induced inflammatory responses [47]. Insulin-like growth factor 1 (IGF-1), considered to be a central biomarker in nutrition and inflammation [48], did increase following selenium supplementation [49].

**Figure 1:**
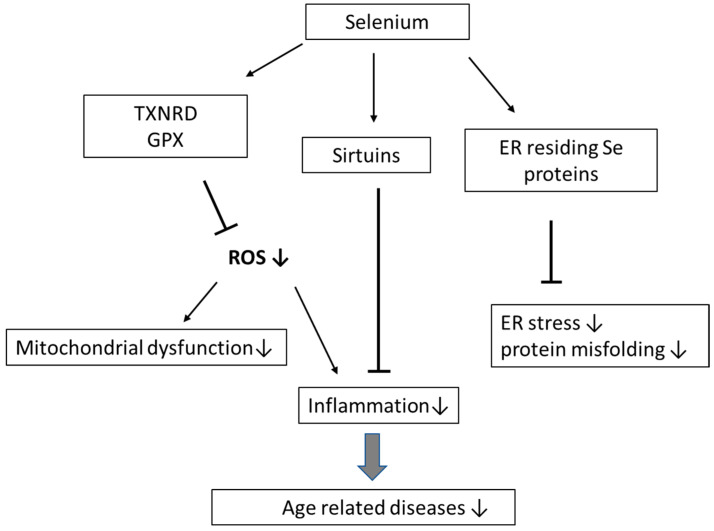
Impact of selenium on excessive production of ROS and inflammation and ageing—potential mechanisms.

The phenomenon of increased inflammatory response in the elderly may in part be related to reduced expression of sirtuins. The family of sirtuins (SIRT-1–SIRT-7), with different cellular localization (nucleus, mitochondria, cytosol), have been associated with longevity. Sirtuin enzymes belongs to class III of histone deacetylases and deacetylate histones and non-histone substrates in a nicotinamide adenine dinucleotide (NAD+)-dependent manner and are thus implicated in the regulation of numerous cellular events including cell cycle control and apoptosis, mitochondrial biogenesis, gene silencing, and genomic stability, thereby mediating longevity [50,51]. Sirtuins are also involved in age-related processes such as inflammatory responses, as well as in the control of oxidative stress responses [52]. It has been reported that SIRT-1 and SIRT-6 located in the nucleus, and SIRT1 translocated to cytosol, exert anti-inflammatory effects by interacting with NF-κB subunits [53]. In a recent study, sirtuin levels in peripheral blood mononuclear cells in a group of elderly individuals with CVD were examined [54], disclosing downregulated SIRT-1, SIRT-5, SIRT-6, SIRT-7, and increased serum CRP values in subjects with low serum Se (Se < 0.75 μmol/L). However, by which mechanism selenium possibly is linked to sirtuins is not known. Further research is needed on the impact of Se on inflammation and its possible role in anti-ageing (Figure 1).

### 3.2. Selenium, Ageing and Cardiovascular Disease (CVD)

The protective function of Se against CVD has been debated. Two early studies reported that low Se status represents a risk factor for myocardial infarction, with increased risk at plasma values below 1 μmol/L (about 80 µg/L) [55,56]. According to the EVA study which included an elderly population in France, a plasma selenium level of 1.1 μmol/L exerted a protective action [8], whereas the suggested optimal plasma selenium for GPX activity is somewhat higher, viz. about 1.2 μmol/L [57]. An inverse association between cardiovascular health and selenium status could be shown in populations with Se intakes below about 60 μg/day [58], while others did not detect deficient selenium status to be a risk factor for myocardial infarction, when populations with higher Se levels (above about 1.0 µmol/L) were investigated [59]. The observed elevated risk of ischemic heart disease among elderly subjects (mean age 63 years) with low serum Se levels (<1.0 μmol/L) in Denmark [56], is in agreement with observations on a German population with serum Se levels of about 0.9 μmol/L [60] and a recent study on an elderly population (>70 years of age) performed in Sweden [61]. The latter study reported a significant increase in cardiovascular mortality in the lowest Se quartiles (<0.7 μmol/L) [62]. In the EURAMIC study (1997), which was a multicenter case–control study including 10 centers in Europe and Israel in 1991–92, Kardinaal et al. [63] found a remarkable inverse relation between the risk of myocardial infarction and toenail Se levels only for the included European center with the lowest Se levels (Germany). The BIOSTAT-CHF prospective observational cohort study, in which patients with worsening heart failure were included, showed that patients deficient in selenium (<70 μg/L, 20.4% of enrolled patients) had worse New York Heart Association (NYHA) class and more severe signs of heart failure and lower quality of life than those with higher plasma selenium. Selenium deficiency was also associated with a higher rate of hospitalization for heart failure or all-cause mortality [64]. SELENOP was determined in the Malmö Preventive Project, a population-based prospective cohort study including 4366 individuals that were followed up for 9.3 (8.3–11) years. The risk of all-cause mortality, cardiovascular mortality, and a first cardiovascular event were all inversely associated with plasma SELENOP concentration [65]. In the United States, physicians reported no significant relation between Se in the serum and the risk of CVD in subjects with plasma concentrations above about 1.0 μmol/l [66]. These observations are essentially consistent with meta-analyses of coronary heart disease and Se [67,68]. Thus, Rees et al. [69] concluded in their meta-analysis that Se supplementation did not reduce cardiovascular mortality, but they admitted to have included most of their patients from the Nutritional Prevention of Cancer (NPC) or the Selenium and Vitamin E Cancer Prevention Trial (SELECT) trials, with a mean baseline intake of about 130 µg/day in males and 90 µg/day in females, which is substantially higher than European levels and well above a risk threshold of around 1.0 µmol Se/L (80 µg/L) in plasma, as discussed above [62]. This conclusion also agree with the lack of effect on CVD mortality in the French SU.VI.MAX study that supplemented subjects with 100 µg Se/d together with vitamin C and E, beta-carotene, and zinc in a cohort with baseline plasma Se above this threshold (mean 1.1 µmol/L) [70]. In contrast, a significantly reduced CVD mortality was obtained in the Swedish KiSel study that supplemented participants with 200 µg Se/d for 4 years in an elderly population (>70 years of age) with mean basic plasma values of about 0.8 µmol/L (67 µg/L) [71]. The reduction in CVD mortality was negatively associated with baseline plasma selenium [62].

### 3.3. Selenium, Ageing and Neurodegenerative Diseases

Among neurodegenerative diseases, Alzheimer’s and Parkinson’s disease share many common features, such as atypical protein assemblies and induced apoptosis, offering hope that similar therapeutic principles can be developed to ameliorate these serious disease entities. Ageing appears to be the most significant risk factor for being affected by these neurodegenerative disorders. Redox stress along with mitochondrial dysfunction has been proposed to represent causative links between ageing and neurodegenerative diseases [72]. As Se compounds are known to defend against oxidative stress, Se deficiency may increase vulnerability to these disorders [73], and elevated ROS levels appeared to play a role in the pathologies of Alzheimer’s and Parkinson’s disease, indicating that antioxidative enzymes can exert protective functions [74].

Alzheimer’s disease (AD) is by far the most common neurodegenerative disorder, with no known effective therapy. Several essential trace elements have been suggested to have key functions in the progression as well as in the protection of the AD development, and Se is probably the most crucial [56]. Se is delivered to brain tissues via the selenoprotein SELENOP, and intraneuronal selenoproteins act as controllers of cellular redox state, calcium homeostasis, protein misfolding, immunomodulators, and regulators of apoptosis [75,76]. The Prevention of Alzheimer’s Disease with Vitamin E and Selenium (PREADVISE) study is a double-blind, randomized controlled trial recruiting 7540 participants. It was transformed into an observational cohort after discontinuation of the SELECT parent trial [77]. The intervention supplements containing selenium and vitamin E were given either alone or combined in addition to a placebo group, but neither supplement prevented dementia. It should be noted that baseline selenium concentrations in the SELECT study were much higher than those common in, for example, Europe (see above). There are epidemiological indications that selenoproteins can protect against cognitive decline [78]. Thus, an Italian cohort investigation in 65–70-year-old people demonstrated that the MMSE (mini-mental state examination) scores, along with performance-based assessment scores of coordination, were substantially decreased in individuals with low concentrations of plasma Se (<66.7 μg/L) [79]. Another cohort trial carried out on French participants aged 60–70 years revealed that cognitive decline was more prominent in individuals with low plasma concentrations of Se (<75.8 μg/L) [80]. In accordance with these observations, Se supplementation offers promising results in terms of improved neuropsychological functions [11,73]. For example, a daily intake of one Brazil nut for six months, which is equivalent to about 250 µg Se/day, stimulated improvement in patients with cognitive decline [81]. However, it should be noted that age-related cognitive decline does not necessarily indicate the diagnosis of AD, and thus, the results of these studies should be interpreted with caution. Nevertheless, the results of a recent meta-analysis showed that decreased levels of Se characterized brain tissues of patients with AD as compared with control brains, which supports the idea of Se supplementation in AD [74].

Parkinson’s disease (PD) is another neurodegenerative disorder with increasing incidence with increasing age. It is characterized by locomotor dysfunction provoked by dopaminergic neuronal death in substantia nigra pars compacta [82]. Oxidative stress appears to also characterize affected brain regions in PD, although it is not clear if this is a cause or a consequence of neuronal loss [83]. Se supplementation has been reported to improve locomotor function in animal models of PD [84]. An inverse relation between mortality rates from PD and the average soil Se concentrations were established for a USA population [85]. However, there is a need for controlled clinical trials before any definite conclusions considering the role of Se in PD can be drawn.

### 3.4. Selenium, Ageing and Cancer

Several epidemiological studies conducted in Europe, the USA, Japan, and China have indicated a significant protective role of Se in malignancies. A recent meta-analysis concluded that Se at recommended daily intakes above 55 μg/day decreased the risk of cancer [86]. In Finland, the implementation of a state program for the elimination of micronutrient deficiencies, including Se fertilizer supplementation, was accompanied by a reduction in cancer mortality [87]. An American study, referred to as the NPC study, including 1312 patients (mean age 63 years) taking 200 μg Se/day decreased the risk of cancer incidence in general by 37%, colorectal cancer by 58%, prostate cancer by 63%, and lung cancer by 48% [88]. Later, the SELECT study, involving 35,500 people (mean age 63 years), did not report these outcomes and intervention with 200 μg Se/day combined with 400 IU vitamin E/day for 5.5 years did not show a positive effect on cancer incidence in general [89]. However, baseline Se levels were above about 1.4 μmol/L (110 μg/L), reflecting baseline intakes above 120 μg/day, presumably explaining that supplementation with extra Se was without protective effect. Among those with a high selenium status, an increased risk of high-grade prostate cancer was even found [90]. Although supplementation was not protective at such high baseline intakes, a recent meta-analysis concluded that Se at recommended daily intakes above 55 μg/day was associated with reduced cancer risk [86]. This study revealed a significant inverse relationship between the risk of cancer after adjusting for body mass index, smoking, age, and Se intake. A reasonable interpretation is that Se levels somewhat higher than the average intakes in Europe could protect against some cancers. Of note, the protective effect in the American NPC trial of Se (200 μg as Se-enriched yeast) was confined to the lower tertile of the cohort that had a pre-treatment plasma Se below 1.3 μmol/L (106 μg/L) [91]. In contrast, no cancer-protective effects were seen in subjects with baseline plasma concentrations above 1.5 μmol/L. A nested case–control study of Japanese-American men [92] found that the inverse association between prostate cancer and serum Se was significant particularly in current and past smokers. In contrast, no association between selenium concentration in plasma and prostate cancer risk—neither stage nor grade—was seen in a nested case–control study using the large European Prospective Investigation into Cancer and Nutrition (EPIC) cohort [93]. In a nested case–control study using the EPIC cohort it was found that low selenium status and low SELENOP in plasma were associated with an increased risk of colorectal cancer [94]. Accordingly, in a recent case cohort study using the EPIC Potsdam cohort (mean age about 50 years), plasma selenium and SELENOP were associated with a decreased risk of colorectal cancer [95]. Additionally, hepatobiliary cancer risk was inversely associated with selenium status and SELENOP concentration in the EPIC cohort [96].

Protection against cancer by supplementation with Se compounds is not expected in populations with an adequate Se status, i.e., blood plasma levels definitively above 1.0–1.2 μmol/L. Thus, the early studies from Finland that disclosed associations between fatal cancers and prediagnostic low blood plasma Se were performed on populations with blood plasma levels as low as 0.6 μmol/L [97]. A review of prostate cancer studies [98] supports the observation of an association between inadequate or low Se status and risk of advanced prostate cancer, with the strongest association being seen in smokers.

It seems likely that a deficient intake of selenium is associated with an increased risk of certain cancers in elderly people, possibly due to less protection against oxidative stress and inflammation. However, the mechanisms of protection are not known, and other explanations have been suggested, based, i.a., on experimental evidence. Hence, the observed anti-carcinogenic effects of some Se species, when supplemented to populations with levels below the threshold of selenoproteome saturation, have been discussed by Rayman et al. [99], who suggested that some methylated metabolites might exert a chemo-preventive action against cancer, presumably by acting through epigenetic mechanisms. Another epigenetic mechanism is the formation of α-keto acid selenium metabolites that effectively inhibit some histone deacetylases [100], targets which have also been used in the pharmaceutical industry [100]. Protection against age-associated inflammation is presumed to play a role in anti-carcinogenic effects [101].

### 3.5. Selenium, Ageing, and Other Age-Related Diseases

Associations between low levels of Se in serum or blood and several other diseases in elderly people have been observed. Ageing increases the incidence of pathological conditions such as neuropathy, and infectious and rheumatic diseases. The elderly often suffer from glomerular or tubular dysfunction and manifest renal failure [102], and there seems to be a close relationship between renal function and Se status [103]. Peritoneal dialysis as well as hemodialysis can lead to a decrease in Se levels in the body [104], and low SELENOP levels appeared to be associated with reduced renal function [105]. In hemodialysis patients, supplementation with Se significantly increased GPX and plasma Se levels, and normalized IL-6 [106].

## 4. Selenium, Genomic Stability, and Telomere Length

**Figure 2:**
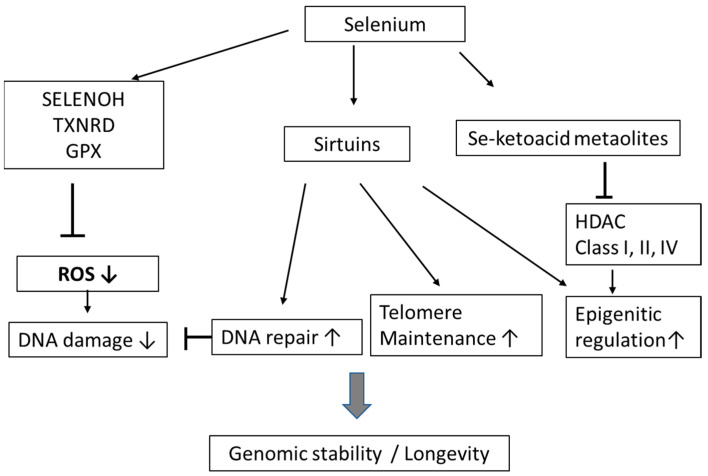
Impact of selenium on genomic stability and ageing—potential mechanisms.

Maintenance of genomic stability, with a low rate of genotoxic events, may prevent age-related decline. Nuclear selenoproteins are thought to play a direct role in gene maintenance via their protection against oxidative stress. Interestingly, selenoprotein H, which is ranked to the highest hierarchy among selenoproteins, is extensively expressed and is localized in the nucleus [107]. Se deficiency and ageing appears to be tightly accompanied by decreased expression of selenoprotein H [108]. Other studies have shown that Se reduces oxidative damage to DNA and prevents DNA adduct formation and DNA breakage [109]. As mention above, selenium status may also be related to expression of sirtuins. Of interest is that SIRT-1, SIRT-2, SIRT-3, SIRT-6 and SIRT-7 are involved in recombinational and base excision DNA repair and maintenance of genomic stability, all important functions related to longevity [110] (Figure 2).

It is well known that the inhibitions of other classes of histone deacetylases (HDAC) (i.e., class I, II, and IV) may delay ageing and promote longevity in experimental systems by, i.a., contributing to the maintenance of histone acetylation and genome stability [111]. As some natural organic selenium compounds can be biotransformed to keto acid HDAC inhibitors [112,113], this might also be a mechanism by which selenium could promote longevity.

Telomeres are protecting caps at the end of chromosomes, and telomeres are regarded as reliable biomarkers for the prediction of ageing. The length of telomeres gradually decreases with the number of times the cell reproduces. Oxidative stress, chronic inflammation, and an unhealthy lifestyle can further increase the telomere shortening rate. Accumulating evidence has demonstrated an association between shorter telomeres and age-related chronic diseases such as CVD, but the direction of causality is not clear [114,115]. Telomere length can to some extent be maintained by the telomerase enzyme, which is composed of telomerase RNA, referred to as TERC, telomerase reverse transcriptase (TERT), and cofactors [116]. Of note, telomerase can only compensate for telomere shortening to a limited extent, and its expression is absent in most somatic cells. Critical short telomeres will not be able to protect DNA from deterioration, and the cells are triggered into replicative senescence, entering apoptosis, which in the end will cause ageing. Accordingly, the maintenance of telomere length emerges is a crucial strategy to retard the ageing process. A defect in the selenoprotein synthetic machinery causing deficient levels of many selenoproteins has been related to shortened telomeres in peripheral blood monocytes [117]. A recent observational study indicated that dietary Se intake was related to longer telomeres in middle-aged and older adults in America [118]. These results were in accordance with observations by Wu et al., who showed that dietary Se deprivation induced telomere shortening in colonocytes of mice carrying humanized telomeres (G3 Terc−/−) [35]. While selenium deprivation in these mice accelerated decline in age-related health, it surprisingly also promoted longevity, in accord with another study in mice [34]. It is tempting to hypothesize that the biological mechanism in the association between dietary Se intake and telomere length is associated with its antioxidant and anti-inflammatory effects. Increased oxidative stress, e.g., caused by tobacco smoking [114], is believed to play a key role in telomere attrition [119]. The authors suggest that a modestly increased Se intake may be beneficial to reduce telomere attrition induced by inflammation and other age-related factors [90]. Of relevance here is that supra-nutritional selenium intake may have pro-oxidative effects [120], which was also reported to be associated with an increased risk of diabetes type 2 [121]. However, there is no reported evidence of adverse effects of dietary organic Se contents up to the tolerable upper intake level of 300 μg Se/d according to European Food Safety Authority (EFSA) [122].

## 5. Concluding Remarks and Future Perspectives

Biological ageing is known as a complex process comprised of molecular heterogeneity, molecular damage, changes in immune function and metabolic imbalance, along with increased susceptibility to diseases and environmental stressors. Numerous studies have reported that the deficiency in Se is closely associated with human ageing and ageing-associated diseases. The related mechanisms are comprised of oxygen-ROS-associated damage, such as genomic instability and telomere shortening, protein misfolding and gene alterations as well as inflammation.

Dietary Se is an important nutritional agent in the protection of age-related diseases also mediated via the immune response. There are numerous biochemical mechanisms in which Se interact with the immune system and Se has the capacity to form a series of different selenoproteins, all of which contain the Sec. However, previous investigations have also provided conflicting evidence, with an apparent absence of a significant relation between Se intake and some ageing-related diseases, such as CVD and cancer. Discrepancies in results and absence of beneficial effects can in many cases be ascribed to the fact that pre-interventional selenium status has not been fully evaluated, and that some studied populations already at inclusion had a good selenium status. Furthermore, as regards carcinogenesis, it seems likely that a deficient intake of selenium with a lack of functional selenoproteins and less protection against oxidative stress and inflammation plays an important role, although it is possible that some Se compounds can possess chemo-preventive characteristics. Consequently, there are ambiguities regarding what is the optimal selenium intake.

Not discussed in this review are possible adverse effects of a high intake of selenium, as there is a rather narrow window of adequate and safe intake. Additionally, the protective mechanisms of Se supplementation, the role of different chemical forms of Se, and which of the aging-associated diseases that are affected by selenium supplementation need to be further clarified.

Finally, further research is highly requested to clarify the proposed protective role of Se when it comes to anti-aging properties and prevention of age-related diseases.

## Data Availability

Not applicable.
